# Repair of an Extensive External Cervical Resorption Lesion Using Intentional Replantation with Crown Rotation

**DOI:** 10.1155/2023/2103999

**Published:** 2023-06-26

**Authors:** Motoki Okamoto, Yoko Asahi, Henry Fergus Duncan, Nanako Kuriki, Yusuke Takahashi, Mikako Hayashi

**Affiliations:** ^1^Department of Restorative Dentistry and Endodontology, Osaka University Graduate School of Dentistry, 1-8, Yamadaoka, Suita, Osaka 565-0871, Japan; ^2^Department of Oral Science and Translational Research, College of Dental Medicine, Nova Southeastern University, Fort Lauderdale, FL, USA; ^3^Division of Restorative Dentistry and Periodontology, Trinity College Dublin, Dublin Dental University Hospital, Dublin, Ireland

## Abstract

Treatment of large external cervical resorption (ECR) lesions may be compromised, rendering the tooth unrestorable. Intentional replantation is a potential treatment option depending on the site and extent of ECR. We present a case of a large ECR successfully managed with intentional replantation with rotation of the tooth. The female patient consulted the hospital clinic, with an extensive palatal ECR on the maxillary lateral incisor. Routine planar radiographs and cone-beam computed tomography demonstrated a larger palatal than the ECR lesion (Heithersay Class III and Patel's Class 2Bp) not amenable to nonsurgical treatment. Intentional replantation after short-term extrusion was planned. The defect was restored, then a palatal ferrule was achieved by rotating the tooth by 180°. At the 18-month follow-up, the periradicular and periodontal tissues remained healthy, and no other symptoms were reported. In conclusion, this successful video-illustrated clinical case highlights the benefits of intentional replantation in saving teeth with advanced ECR.

## 1. Introduction

External cervical resorption (ECR) is a form of root resorption that results from the activation of osteoclasts causing loss of cementum and dentine [1]. The clinical presentation of ECR varies but is often asymptomatic, with the differential diagnosis, including internal resorption, cervical caries, and root surface caries. The aetiology of ECR has been attributed to several factors, including trauma and orthodontic treatment; however, the exact cause is often unknown, and the disease pathogenesis remains poorly understood. It is therefore important to publish descriptive case reports and conduct further clinical studies to assess treatment options and highlight limitations.

Although ECR can occur in any type of tooth, the maxillary anterior teeth (incisor and canine) are most commonly affected [2]. Previously, ECR classifications were based on information from two-dimensional (2D) periapical and panoramic radiographs, which subsequently guided the treatment plan [3]. Although classification using 2D methods can be problematic, particularly for molars, they still play an important role in the preliminary treatment planning for ECR. Recently, cases of ECR with different aetiologies, including medications have been reported [4, 5]. In addition, histopathological, cone-beam computed tomography (CBCT), micro-CT, nano-CT, and scanning electron microscopy have revealed different pathogenesis mechanisms [6–8]. The clinical application of CBCT in a three-dimensional (3D) classification was recently reported to play an important role in determining the subsequent treatment plan [9], and its use in addition to the conventional 2D classification was proposed. In 2018, the European Society of Endodontology published a position statement on ECR that summarised contemporary knowledge and provided recommendations for clinical practice [10]. In recent years, several cases of ECR have been reported, with lesions of various sizes at different stages of progression and successful management with the recommended treatment methods [11, 12]. Intentional replantation, which is considered the most invasive treatment option for ECR, is a potential treatment strategy based on the degree of ECR. It provides a high probability of restoration of the destroyed tooth structure [13]. Multiple case reports detailing the treatment of ECR have shown the efficacy of endodontic and periodontal approaches alone or in combination [14–16]. Although there are only a few case reports of intentional replantation, some case reports are available demonstrating the efficacy of intentional replantation for ECR [4, 17]. Intentional replantation should involve minimal damage to the periodontal ligament and ideally be combined with surgical extrusion and other procedures that enable future crown restoration and improve the predictability of the restorative treatment. In particular, hard tissue destruction due to advanced ECR on the palatal aspect of anterior teeth can be closely related to the long-term effects of dental restorations due to the nature of the constant tensile stress related to occlusal forces. Here, the management of a maxillary lateral incisor with a large ECR lesion is described. The lesion was treated by temporarily subjecting the tooth to orthodontic forces to minimise the damage to the periodontal ligament, extracting the tooth, removing the ECR under a dental operating microscope (DOM), and replantation of the crown after 180° rotation.

## 2. Case Description

**Figure 1:**
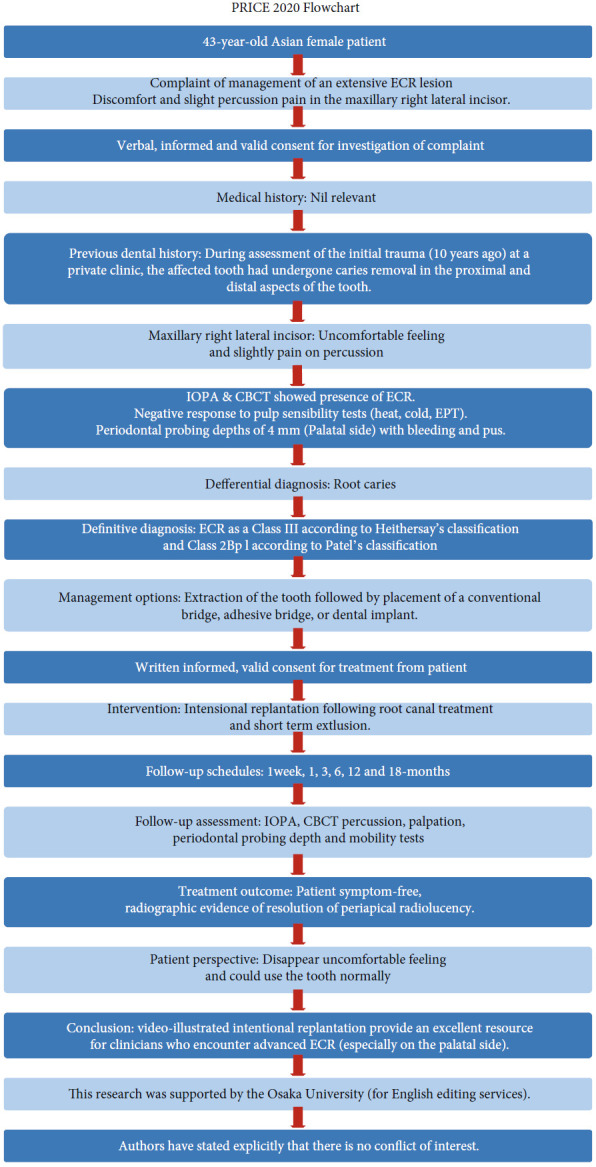
PRICE 2020 flowchart showing the steps involved in the case report. ECR, external cervical resorption; CBCT, cone-beam computed tomography; EPT, electric pulp testing; IOPA, intraoral periapical radiograph.

This case report has been written according to Preferred Reporting Items for Case Reports in Endodontics (PRICE) 2020 guidelines [18]. The PRICE 2020 flowchart was shown in Figure 1. Informed consent has been obtained from the patient for publication as a case report of the process of treatment for this ECR lesion. An overview of the treatment timeline is presented in Table 1. A 43-year-old non-smoking female was referred to our university hospital clinic by a private dental clinic for the management of an extensive ECR lesion. The patient complained of discomfort in the maxillary right lateral incisor and reported a history of a fall that happened 10 years ago. However, the patient had not paid particular attention at the time, as the patient was asymptomatic immediately after the trauma, and there was a lack of superficial damage. During the assessment of the initial trauma (10 years ago) at a private clinic, the affected tooth had undergone caries removal in the proximal and distal aspects of the tooth. Several years later, the patient was diagnosed with ECR at a private dental clinic. The discomfort at the affected tooth gradually increased, and the radiographic translucency of the ECR lesion became larger.

At the time of presentation, the patient was not on any medications and had no relevant general medical or family history. Furthermore, common causes of ECR, such as history of orthodontic treatment, parafunctional habit, or abnormal occlusal pattern, were not detected. Overall, oral hygiene was good, and plaque levels were well controlled. Bruxism or occlusal interference was not apparent. There was no pathological tooth mobility of either the affected or its adjacent teeth. No tenderness was noted on palpation, and slight pain was reported on vertical percussion. Pulp sensibility tests demonstrated a negative response to thermal stimulation (cold test; Pulper, GC, Tokyo, Japan) or the electrical pulp test (Digitest II, MORITA, Kyoto, Japan) in comparison with the responses to the adjacent teeth. The periodontal probing depth was less than 3 mm on the buccal side, but 4 mm deep, with bleeding and slight pus exudate from the distal aspect of the palatal surface. Root caries as a differential diagnosis were ruled out because no infected dentine was detected.

**Figure 2:**
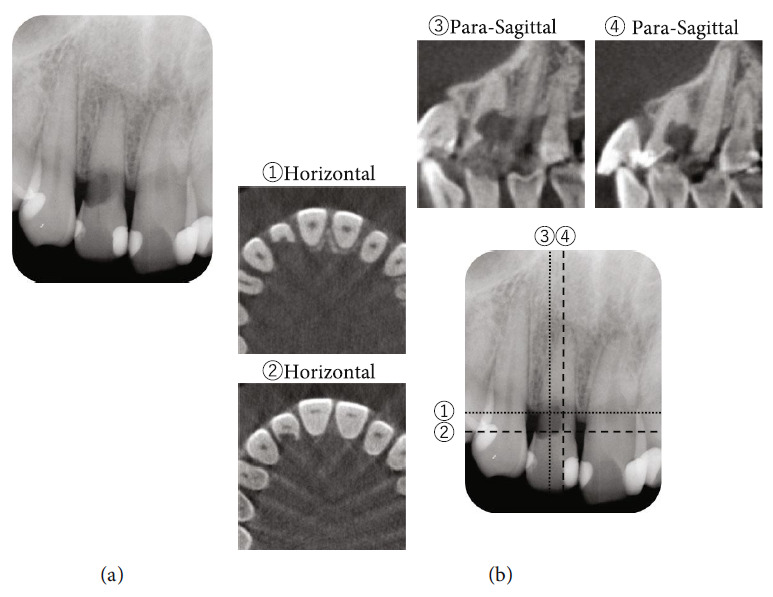
Radiographic images taken during the patient's initial visit to the University Clinic. (a) Intraoral radiographic image. A large translucency was suggestive of ECR at the maxillary right lateral incisor, extending from the distal to the mesial side, can be observed. It was difficult to accurately determine the buccal-to-palatal extent of ECR. The pulp chamber appeared narrowed adjacent to the ECR lesion. (b) Selected horizontal and para-sagittal images of CBCT volume. 3D observation by CBCT shows that external resorption of the cervical area of the tooth was more pronounced on the palatal side and was diagnosed as a type 2Bp according to Patel's classification. The numbers given in the figure indicate a cross-sectional view of each line of that number.

An intraoral radiograph (Figure 2(a)) revealed a large ECR lesion extending from the distal to the proximal side; however, no obvious radiolucency was evident at the root apex. No radiographic signs of swelling or sinus tract were observed at the right maxillary incisor. CBCT was performed to further explore the extent of the ECR (Figure 2). Horizontal and sagittal CBCT images showed that the ECR lesion had reached the pulp tissue in up to one-third to one-half of the root in the axial direction, and horizontally, the hard tissue defect extended by approximately 180° mainly on the palatal side. It was accordingly diagnosed as a Class III according to Heithersay's classification [3] and Class 2Bp lesion according to Patel's classification (Figure 2(b)) [9]. After diagnosis and classification, a treatment plan (including options) was discussed with the patient. The patient was specifically informed that due to the extent of ECR, it would be difficult to perform root canal treatment (RCT) without accompanying periodontal surgery, which would also include debridement and restoration of the destructed hard tissue.

Owing to the patient's desire to retain the tooth after explanation, consent was obtained for intentional replantation after RCT. Extraction of the tooth followed by placement of a conventional bridge, adhesive bridge, or dental implant (immediate or delayed) was discussed as a potential alternative to intentional replantation. ‘Ideal' rubber dam isolation clamping the tooth in question was not possible due to a lack of coronal tooth substance so a split dam technique was all that could be used if RCT and intentional replantation were selected. After presenting the advantages and disadvantages of each treatment method to the patient, the placement of an adhesive bridge after tooth extraction was considered only if intentional replantation was unsuccessful.

**Figure 3:**
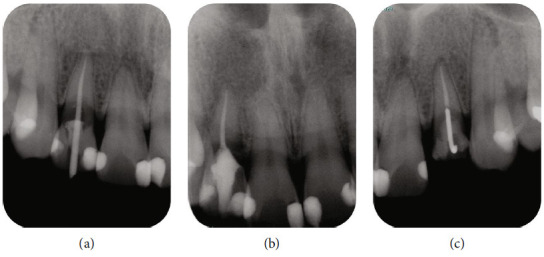
Radiographic images before intentional replantation. (a) Endodontic treatment was performed on the maxillary right lateral incisor after the pulp sensibility test result was negative. The working length was confirmed using a gutta-percha cone. (b) Intraoral radiographic image was taken immediately after the root canal filling with a single cone method using a methacrylate resin sealer (MetaSEAL soft paste). (c) An orthodontic hook was placed to assist intentional replantation with minimal damage to the periodontal ligament.

Based on the clinical examination, ECR treatment was carried out under magnification using a DOM (M320-D, Leica Microsystems, Wetzlar, Germany). Due to the nature and extent of the ECR lesion, as discussed previously the split dam isolation technique was performed under local anaesthesia, and the palatal gingival tissue adjacent to the ECR was removed using an electronic scalpel under dry conditions to facilitate coagulation. Thereafter, RCT was initiated using a diamond bur with a water-cooled high-speed handpiece. The root canal was narrowed due to ECR and required careful negotiation to access the root canal system. Apical patency was maintained with an H-file #10 (Dentsply Sirona, Ballaigues, Switzerland), and root canal length was measured electrically using an apex locator (Root ZX3, MORITA). Subsequently, a glide path was established, and the root canal was enlarged using a nickel-titanium (NiTi) file system (HyFlex EDM, Coltene, France) with an Endomotor (TriAuto ZX2, MORITA), which was capable of simultaneously measuring the electrical root canal length. The working length was further confirmed on a periapical radiograph image by placing a gutta-percha cone (Figure 3(a)). The root canal was profusely irrigated with 2.5% sodium hypochlorite (Neo Cleaner, NC, Neo Dental Chemical Products Co., Ltd., Tokyo, Japan) and 3% ethylenediaminetetraacetic acid solution (Smear Clean, Nishika, Yamaguchi, Japan) delivered using a 27-G root canal irrigation syringe (Neo Dental Chemical Products Co., Ltd.) with simultaneous agitation using a NiTi instrument (X-p Endo finisher, FKG Dentaire, La Chaux-de-Fonds, Switzerland). The root canal was then dried with sterile paper points. A calcium hydroxide dressing (Calcipex II, Nishika) was placed. The first-visit treatment was completed by carefully placing a temporary restoration of glass ionomer cement (GlassIonomer FX ULTARA, Shofu, Kyoto, Japan) up to the root canal orifice to avoid coronal leakage from the palatal side.

**Figure 4:**
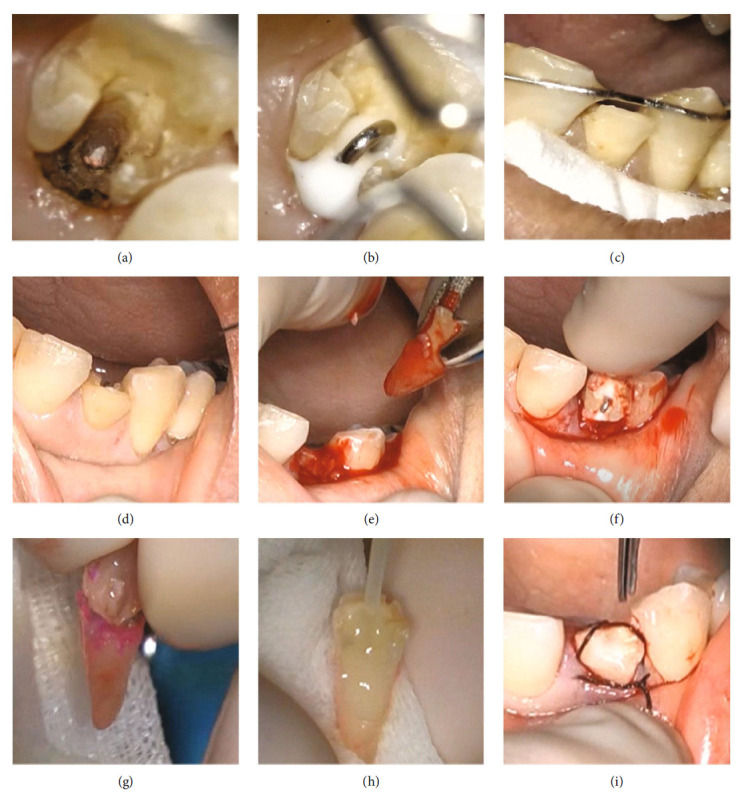
Clinical images of the custom orthodontic hook used for extrusion and intentional replantation. Videos are available online at https://doi.org/10.6084/m9.figshare.20787508.v1 and https://doi.org/10.6084/m9.figshare.20787613.v1. (a) After root canal filling, the gingiva of the ECR lesion on the palatal side was removed with an electro-scalpel to access the hook for extrusion. (b) A question mark-shaped bending Co–Cr wire was placed in the root canal with carboxylate cement. (c) Bent Co–Cr wires were also bonded to both adjacent teeth with 4META-MMA/TBB resin. (d) The extrusion hook was removed immediately before intentional replantation. (e) Tooth extraction was carried out with forceps. (f) Root fit in the extraction socket was confirmed after crown rotation. (g) The ECR area was detected using a red dye (caries detector) marketed for caries treatment and visualized using a DOM. (h) A resin-based composite construction with a fibre post was used. (i) After the extraoral procedure, the rotated lateral incisor was replanted.

At the second RCT visit, the tenderness on percussion had significantly subsided. The glass ionomer cement and calcium hydroxide dressing was removed after isolation using a split dam rubber dam technique, and the root canal was re-irrigated and dried. Then, the root canal was filled with a methacrylate resin sealer (MetaSEAL soft paste, Sun Medical Co. Ltd., Shiga, Japan) and a single-matched taper gutta-percha cone (Figure 3(b)). As the tooth was asymptomatic after RCT, an orthodontic hook device was placed for extrusion before intentional replantation (Figures 4(a), 4(b), and 4(c)). A video illustrating these processes is provided as video information (refer to https://doi.org/10.6084/m9.figshare.20787508.v1). After administering local anaesthesia, the temporary sealing material was removed, and the gingival tissue on the palatal side of the ECR was kept dry by removal and coagulation using an electrical scalpel. A 0.7-mm in diameter cobalt–chromium (Co–Cr) hook device was affixed in the root canal using carboxylate cement (HY-BOND Carbo Cement, Shofu), and then a custom-made wire was bridged to the adjacent teeth and fixed with 4-methacryloxyethyl trimellitate anhydride/methyl methacrylate tributylborane (4META-MMA/TBB) resin (Superbond, Sun Medical Co. Ltd., Shiga, Japan). One orthodontic thread was used to tie the wires to provide a sustained extrusion force. Follow-up procedures involved changing the orthodontic threads once per week for three weeks.

The orthodontic device used to extrude the tooth was removed under local anaesthesia before intentional replantation. Intentional replantation was performed by one operator and two assistants in an operating room under magnification (Figures 4(d), 4(e), 4(f), 4(g), 4(h), and 4(i), refer to videos https://doi.org/10.6084/m9.figshare.20787613.v1). The mobility of the tooth increased after device removal, and gentle extraction was performed using forceps (Claw, YDM, Tokyo, Japan). Next, the crown was rotated 180° to confirm that it fits into the extraction socket. This permitted the ECR lesion observed under magnification, and a dye detection solution (usually used for caries detection, Caries detector, Kuraray Noritake Dental Inc., Tokyo, Japan) was utilised to identify the extent of the ECR. The lesion was then meticulously curetted to ensure that no resorptive tissue remained. A resin-based composite restoration was placed and cured using blue light irradiation (Pen cure 2000, MORITA) before the tooth morphology was adjusted. Finally, the tooth was replanted in the extraction socket. A sterile saline solution was used to minimise dryness and contamination of the dentine debris, except during ECR removal and morphological modification of composite restorations using a high-speed handpiece. The crown was sutured to the gingiva to promote initial wound healing and stability and fixed to the adjacent tooth with composite resin for reinforcement. The extraoral work time for the intentional replantation procedure was approximately six minutes. These treatments were performed by an endodontist with >10 years of experience.

**Figure 5:**
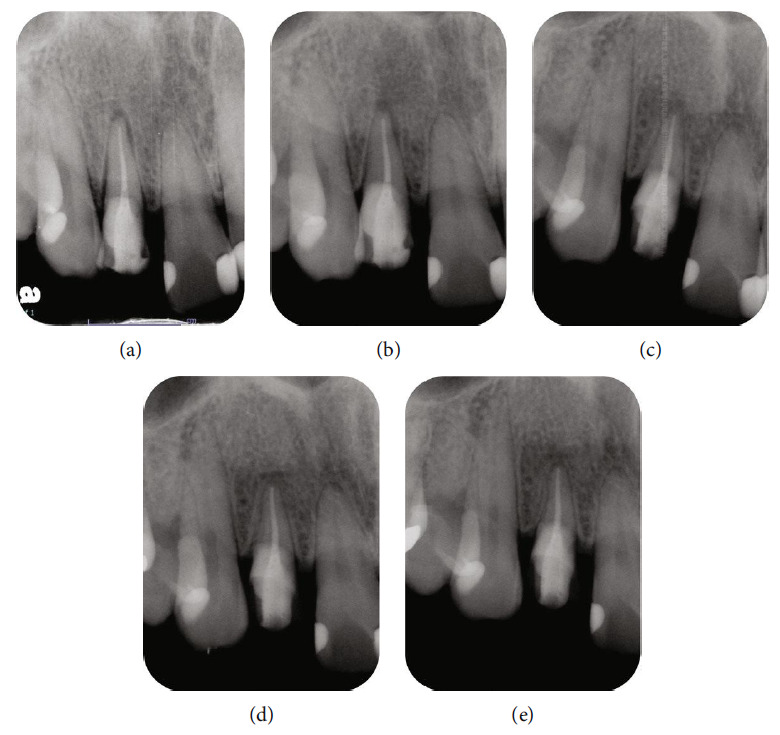
Radiographic images after intentional replantation. (a) Intraoral radiographic image was taken immediately after intentional replantation with crown rotation. (b) Intraoral radiographic image was taken one month after intentional replantation. The periodontal ligament space appears to be slightly larger than that immediately after intentional replantation (c) Intraoral radiographic image was taken three months after intentional replantation. The periodontal ligament space is similar to that around the adjacent teeth. (d and e) Intraoral radiographic images were taken six months and one year after intentional replantation, respectively. Low-contrast component resin crowns are bonded with adhesive resin.

**Figure 6:**
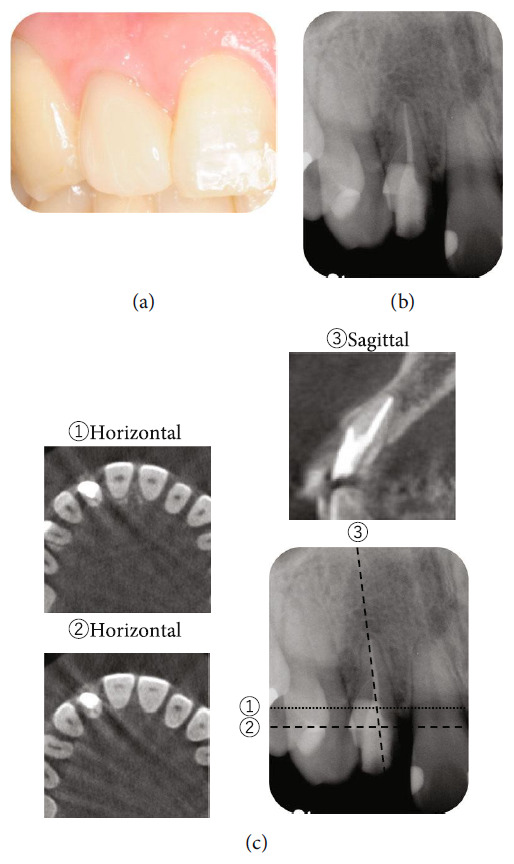
Follow-up visit 18 months after intentional replantation. (a) Intraoral photograph was taken 18 months after intentional replantation. No gingival redness or swelling was observed. (b) Intraoral radiograph was taken 18 months after intentional replantation, with no evidence of recurrence of ECR or secondary root resorption after intentional replantation. (c) Representative examples of horizontal and sagittal images of CBCT volumes; 3D evaluation by CBCT showed that the ECR lesion was now filled by the restorative material, and good healing was observed. The numbers in the figure indicate the cross-sectional view of each line of that number.

Follow-up appointments were scheduled at various time points (Figures 5(a), 5(b), 5(c), 5(d), and 5(e)). After one week, the sutures and resin cement were removed. Three months after intentional replantation, a provisional restoration was fabricated. RCT was performed with the split dam isolation due to the risk of periodontal exudate contaminating the canal. The tooth was observed for a prolonged period after obturation, with no subsequent symptoms or abnormal findings evident clinically or radiographically. After determining that additional root canal retreatment was not necessary based on a 6-month follow-up, a composite resin crown (Kuraray Noritake Dental Inc.) was constructed using computer-aided design/computer-aided manufacturing as the final step in the restoration process. There were no clinical symptoms during this period, and radiographs showed no detrimental effect of intentional replantation. Furthermore, no clinical symptoms or abnormal findings were observed 18 months after intentional replantation. These results demonstrate the success of ECR treatment with RCT and intentional replantation (Figure 6).

## 3. Discussion

The accumulation of scientific knowledge regarding ECR enabled by the recent widespread use of CBCT and the publication of position statements by the European Society of Endodontology has led to increasing awareness regarding the condition [10]. The closely related aetiological factors of ECR include orthodontic treatment and previous traumatic injury. In addition, factors detrimental to the periodontal ligament, such as parafunctional habits, malocclusion, and bleaching agents for discoloured teeth, may also be involved [13]. The roles of specific bacteria and viruses, genetic predisposition, and systemic diseases and their medications have also been discussed. Since the lesion in the present case was limited to one maxillary lateral incisor, the traumatic injury that had occurred 10 years ago was the most likely cause of the ECR. Regular visits to the dentist are important for early detection of the lesion, which patients are usually unaware of. If ECR is detected early, curettage and restoration of the defect with a resin-based composite or glass ionomer cement can be performed after periodontal surgery. If the tooth is non-vital, restoration with biocompatible sealing and high-sealing materials, such as hydraulic calcium-silicate cement, can be performed after RCT. Depending on the extent and progression of ECR, further treatment, including intentional replantation or tooth extraction, may be required [19].

The present case was of a hard tissue defect extending from one-third to one-half of the root length on the palatal side and pulp. It was diagnosed as a Class III lesion based on Heithersay's classification and as a Class 2Bp lesion based on Patel's classification (Figure 2). The clinical and radiographic findings indicated that the successful management of this advanced ECR defect was difficult. Therefore, although intentional replantation is a reliable treatment for ECR, crown or root fracture during tooth extraction, or damage to the periodontal ligament during removal of the ECR-affected dentin debris, whereas removing the granulation tissue or during restorative material adhesive manipulation, may result in external resorption or ankylosis may occur. These risks were conveyed to the patient, and it was explained that if this treatment method did not work, an adhesive bridge would be placed after tooth extraction. RCT was necessary before replantation as the pulp was necrotic. A component of the pathogenesis of ECR is root resorption resulting in dental and local hard tissue loss [20]. In the present case, as ECR progressed, there was sclerosis and narrowing of the pulp, making it difficult to locate the root canal during RCT. The DOM and CBCT images were highly effective in visualising the narrowed pulp space. Although rubber dam isolation is essential for RCT, placement of the clamp was difficult due to the palatal ECR defect. Therefore, the split dam isolation technique was used with a clamp applied to both adjacent teeth. However, even with this method, complete isolation was not possible due to the presence of granulation tissue palatal to the defect. Therefore, the gingival tissue on the palatal side was removed with an electrical scalpel under local anaesthesia to prevent contamination with saliva and blood, whereas the DOM was used to constantly monitor the wound. Although root canal filling was performed on confirming a dry intracanal using the DOM, we explained to the patient that a second reRCT would potentially be performed under single tooth rubber dam isolation if negative signs appeared during the follow-up after intentional replantation. These suggestions were based on the patient's desire to save the tooth and the patient's preference for the number of treatments, and the patient fully agreed. Following RCT and before intentional replantation, a custom-made orthodontic appliance was fabricated to minimise the damage to the periodontal ligament caused by tooth extraction (Figures 4(a), 4(b), and 4(c)). This orthodontic hook device was placed for three weeks to apply sufficient force on the orthodontic threads. Basic science research studies have shown that the periodontal ligament on the traction side is activated with accompanying gene expression of GDF15, which is related to wound healing of the periodontal ligament and may be advantageous for replantation [21]. A clinical study showed that applying an extractive force for 2–3 weeks before intentional replantation can improve the prognosis of the tooth [22]. Moreover, extraction instruments that minimise the stress on the periodontal ligament have also been developed [23]. In the present study, extraction was easily performed due to existing tooth movement upon removal of the device.

Mechanical or chemical treatment of the ECR site, using trichloroacetic acid or sodium hypochlorite, is recommended [24]. However, this was not done in the present case to minimise damage to the periodontal ligament as periodontal ligament integrity influences prognosis. Instead, there was a focus on effective mechanical removal of the lesion under a large field of view using the DOM and enhancement with dye staining, which effectively facilitated an accurate dental procedure. Recently, video case reports have become available with more detailed and specific treatment methods, thus clinical tips can be accumulated from the magnified view video presentations [25]. Extraoral working time is another consideration for preventing periodontal ligament damage. It has been reported that the prognosis is better when the extraoral working time is ≤15 minutes [26, 27]. In the present case, the extraoral working time was only 6 minutes, which suggests that the damage to the periodontal ligament was minimised. In maxillary anterior teeth, the palatal aspect of the tooth is affected by occlusal stress, and the creation of a palatal ferrule after endodontic treatment has been recommended to limit fracture resistance [28]. CBCT images can reveal the subgingival placement of ECR restorations at the level of the alveolar bone. Therefore, the composite resin crown margins do not reach the sound dentine, which means that approximately a quarter of the root a ferrule is not achieved. However, it is easy to achieve a ferrule on the labial aspect. Periodontal disease could be expected on the palatal side as supracrestal margins were not secured, but as shown in Figure 5(a), there was no evidence of gingivitis.

No periodontal symptoms were observed in an 18-month follow-up period; however, the long-term effects need to be monitored. There is a higher likelihood of tooth fracture or alveolar bone resorption as a ferrule was not achieved. To our knowledge, no case or clinical study of ECR restoration with crown rotation and replantation has been reported. Therefore, reporting and accumulating similar cases is important. Negative sequelae after intentional replantation occur in most cases within one year [27]; however, long-term follow-up is necessary to accurately assess treatment outcomes. In the present case, there were no clinical symptoms, and no external resorption or other pathology was observed at 18 months. Although continued follow-up will be necessary in the future, the case can be considered clinically successful at this stage.

In the presence of an extensive ECR affecting a maxillary incisor as illustrated in this case report, several treatment options are available. Concerning the cost-benefit of the selected treatment, extraction, and adhesive bridge or implant placement are also options for the patient. Extraction and adhesive bridge placement limit the number of appointments for the patient and are simple to do compared with an implant; however, implants do not affect the adjacent teeth. Unfortunately, at present, there has been little formal research addressing the cost-benefit analysis of treating advanced ECR lesions. Intentional replantation was the only way to save the tooth as was the patient's wish. It required endodontic, oral surgery, and orthodontic knowledge and skills, but no special equipment. These are demonstrated in the DOM video. Intentional replantation is justified if the visiting number and duration of treatment are acceptable to meet the patient's wishes but is likely to cost a similar amount to adhesive bridge provision albeit without the need for laboratory assistance.

## 4. Conclusion

In the present case, an extensive ECR lesion, potentially caused by trauma received 10 years ago, was observed using radiographic images. Tooth preservation was achieved through RCT and intentional replantation. Although intentional replantation is considered the most invasive treatment method for ECR, relevant reports, including video-illustrated case reports like this, can demonstrate the successful performance of technique-sensitive procedures and provide an excellent resource for clinicians who encounter advanced ECR (especially on the palatal side) in their practice.

## Figures and Tables

**Table 1:** Timeline symptoms and treatment of maxillary second incisor.

	Clinical main symptoms	Clinical treatment
Ten years previously (private dental clinic)	Asymptomatic	She visited a private clinic due to trauma. Caries treatment
Several weeks previously (private dental clinic)	Uncomfortable feeling	Referred to a university hospital for ECR
	Radiographic translucency became larger	
Root canal treatment (at dental hospital)	Uncomfortable sensation	Root canal treatment
	Slightly tenderness to the percussion	
	No tenderness to palpation	
	Pulp sensibility tests show negative (thermal stimulation test and electrical pulp test)	
	Periodontal probing depths of 4 mm (palatal side) with bleeding and slight pus	
	No pathologic tooth mobility	
Root canal filling (at dental hospital)	Resolution discomfort	Root canal treatment (root canal filling)
	No tenderness to percussion and palpation	
	No periodontal problems	
Orthodontic treatment (at dental hospital)	No clinical symptom	A custom orthodontic device was set (3-week duration)
Intentional replantation (at dental hospital)	No clinical symptom	Granulation tissue and affected ECR dentin were removed.
		Resin composite restoration for defect due to ECR
		Intentional replantation with crown rotation
		The healing period is 3 months due to COVID-19
Follow-up 1-week after intentional replantation (at dental hospital)	Postoperative slight tooth mobility	Removing sutures and fixing resin cement for stability
Follow up 3 months after intentional replantation (at dental hospital)	Postoperative tooth mobility is within normal limit	Provisional restoration was fabricated. (Delayed schedule due to COVID-19)
Follow up 6 months after intentional replantation (at dental hospital)	No clinical symptom	A composite resin crown was constructed
Follow up 18-months after intentional replantation (at dental hospital)	No clinical symptom	

## Data Availability

The original contributions presented in the study are included in the article/supplementary material, further inquiries can be directed to the corresponding author/s. The clinical video data used to support the findings of this study have been deposited in Figshare (https://doi.org/10.6084/m9.figshare.20787508.v1 and https://doi.org/10.6084/m9.figshare.20787613.v1).
